# The Right to Use Celluloid

**Published:** 1875-05

**Authors:** 


*The Right to Use Celluloid.*—The following letter was recently received from a dental practitioner of this city :

" I have in my posession a communication from Josiah Bacon saying : ' Permit me to state that the use of *Celluloid* is an infringement on our Patents ;' by this, I presume he means that if we as dentists use *Celluloid* for dental purposes, we are held responsible for any license he may see fit to inflict upon us. If you see fit, publish this in your next issue of the Journal, as my desire is to have the matter brought out before the dental profession, that we may act as one man in exterminating a great monopoly whose venom is more deadly than the famous Vampire."

In answer to the above letter, we will state, that so far as we know or believe, the Goodyear Company have no claim whatever on *Celluloid*, or its use as a base for artificial teeth, and in this opinion we are sustained by Dr. S. S. White, who is the agent for the sale of this material. Dr. White speaks as follows : " I am not aware that Bacon has obtained a ' controlling' or any other interest in the celluloid base, and as I am the *sole agent* for its sale, I am sure such a thing could not happen without my knowledge."

Many practitioners after using *Celluloid* for a long time, express their satisfaction with it, and their belief in its durability.

